# UK guidance on the initial evaluation of an infant or an adolescent with a suspected disorder of sex development

**DOI:** 10.1111/j.1365-2265.2011.04076.x

**Published:** 2011-07

**Authors:** S Faisal Ahmed, John C Achermann, Wiebke Arlt, Adam H Balen, Gerry Conway, Zoe L Edwards, Sue Elford, Ieuan A Hughes, Louise Izatt, Nils Krone, Harriet L Miles, Stuart O'Toole, Les Perry, Caroline Sanders, Margaret Simmonds, A Michael Wallace, Andrew Watt, Debbie Willis

**Affiliations:** * Royal Hospital for Sick Children, University of GlasgowGlasgow; † Developmental Endocrinology, UCL Institute of Child Health, University College LondonLondon; ‡ Institute of Biomedical Research, University of BirminghamBirmingham; § Leeds Centre for Reproductive Medicine, Leeds Teaching HospitalsLeeds; ¶ Department of Endocrinology, University College London HospitalsLondon; ** Alder Hey Children's NHS Foundation TrustLiverpool; †† CLIMB CAH Support GroupCambridge; ‡‡ Department of Paediatrics, University of CambridgeCambridge; §§ Clinical Genetics Dept, Guys HospitalLondon; ¶¶ Dept Clinical Biochemistry, Barts and the London NHS TrustLondon; *** Department Paediatric Urology, Alder Hey NHS Foundation TrustLiverpool; ††† AIS Support GroupGlasgow, UK; ‡‡‡ Department of Biochemistry, Glasgow Royal InfirmaryGlasgow, UK; §§§ Society for EndocrinologyBristol, UK

## Abstract

It is paramount that any child or adolescent with a suspected disorder of sex development (DSD) is assessed by an experienced clinician with adequate knowledge about the range of conditions associated with DSD. If there is any doubt, the case should be discussed with the regional team. In most cases, particularly in the case of the newborn, the paediatric endocrinologist within the regional DSD team acts as the first point of contact. The underlying pathophysiology of DSD and the strengths and weaknesses of the tests that can be performed should be discussed with the parents and affected young person and tests undertaken in a timely fashion. This clinician should be part of a multidisciplinary team experienced in management of DSD and should ensure that the affected person and parents are as fully informed as possible and have access to specialist psychological support. Finally, in the field of rare conditions, it is imperative that the clinician shares the experience with others through national and international clinical and research collaboration.

## Introduction and development of guidance

Disorders of sex development (DSD) are a wide range of conditions with diverse pathophysiology^1^ that most often present in the newborn or the adolescent. Affected newborns usually present with atypical genitalia whereas adolescents present with atypical sexual development during the pubertal years. These clinical situations can often be difficult to manage, particularly in those cases where the sex of rearing is uncertain. Establishing a dialogue and building rapport with the affected child and the parents, evaluating the child and then developing a logical, as well as pragmatic, plan for investigations are central to the initial approach and ongoing management.

The consensus that was reached in Chicago in 2005 on the general principles of managing patients with DSD represented a historic milestone for international and multidisciplinary collaboration in this area.^2^,^3^ Some areas of care such as the initial approach to evaluating the infant or young person with suspected DSD were not covered in detail as that was beyond the scope of the exercise. Guidance on the initial evaluation of a complex condition is often coloured by local provision of health care, and it was felt that reaching a consensus at a national, United Kingdom, level may be the most effective means of proceeding further. A UK DSD taskforce was formed in March 2009 under the auspices of the Society for Endocrinology, and it was agreed that the remit of this group would be to concentrate on guidance on the initial evaluation process and the diagnostic approach rather than the clinical management of the condition once a provisional or definitive diagnosis has been reached. This guidance would be aimed at a range of clinical professionals who encounter newborns and adolescents with DSD and its purpose would not be to simply act as a manual but to harmonize good clinical practice. Stakeholder professional societies and clinical professionals who could represent these societies were identified and approached to join the group as were members of two patient representative groups. These group members took responsibility for individual sections and based their opinion on observational studies and expert opinion and the whole group considered each draft. After completion, the guidelines were subject to open external review by the involved professional societies and their members as well as patient group representatives. After this period of open consultation of over 3 months, comments were reviewed and most suggestions were incorporated. All members of the group have approved the final draft of the guidance.

## The multidisciplinary team

Optimal care for infants and adolescents with DSD requires an experienced multidisciplinary team (MDT) that should be accessible through regional centres. The team may exist as a clinical network with links between more than one specialist centre. As a minimum standard, the clinical team should include specialists in endocrinology, surgery and/or urology, clinical psychology/psychiatry, radiology, nursing and neonatology. For infants with suspected DSD, this team should develop a plan for clinical management with respect to diagnosis, sex assignment and treatment options before making any recommendations to the parents. In addition, the clinical team should have links to a wider MDT that consists of specialists from adult endocrinology, plastic surgery, gynaecology, clinical genetics, clinical biochemistry, adult clinical psychology, social work and, if possible, to a clinical ethics forum (Table 1).^4^ Ideally, discussions with the family are lead by one professional, and in most situations, particularly in the case of the newborn, the endocrinologist assumes the role of clinical lead and oversees the timely involvement of other members of the team. However, the core composition of the team for each affected person and family will vary according to DSD type, family need or parent preference, local resources, developmental context and location as well as the age of the person. The parents and the young person should be informed of the range of help that is available from the MDT and should be provided with contact details of these personnel. Ongoing communication with the family's primary care physician is important, and consent issues in relation to sharing information outside of the hospital setting should be discussed with parents and young person. The team has a responsibility to educate other healthcare staff and should have a forum to meet regularly, in the context of a clinic and an educational meeting where it can review and discuss its own performance. Audit of clinical activity, research studies, building collaborative working partnerships with other DSD teams and attendance at joint clinics and education events are crucial if knowledge and information sharing is to be optimized across MDT teams. Transfer of care for the adolescent should be organized with the MDT operating in an environment comprising specialists with experience in adolescent care.^5^

**Table 1:** The clinical members of the MDT and their roles in providing care to the patient and the parents. Professionals marked with an asterisk are core members of the MDT who should meet regularly to discuss cases in a clinic setting

Role
Neonatologist or General Paediatrician
Initial explanation
Management of the unwell child
Initiation of first-line investigations
Seek advice from paediatric subspecialist (endocrine or surgical) with an interest in DSD
Paediatric Endocrinologist*
Detailed explanation over multiple visits
Management of the unwell child
Interpreting first-line investigations and planning second-line investigations
Organize timely and appropriate involvement of other members of MDT
Act as the link between the parents and MDT
Initiate and monitor long-term medical therapy such as steroid or sex steroid therapy
Paediatric Radiologist
Interpret and often perform ultrasound scans in the newborn
Judge the reliability of ultrasound scans in the newborn especially when the results may influence sex assignment
Assessment of external anatomy
Paediatric Urologist*
Explanation of the anatomy and results of imaging
Explanation of pros and cons of reconstructive surgery
Develop a plan for complex imaging (other than pelvic ultrasound) and further assessment of the anatomy
Perform procedures such as laparoscopy, biopsy, reconstructive surgery and gonadectomy
Organize timely and appropriate involvement of other members of MDT
Paediatric Specialist Nurse*
Provide general support to the patient and parents in addition to that provided by other members of the MDT
Arrange specialist investigations
Liaise with the rest of the DSD team, especially the clinical psychologist
Clinical Psychologist*
Provide specialist support to parents soon after birth
Provide support to the growing child and the parents
Develop an individualized plan for each family
Guide the MDT on timing and tempo of explanation of the condition to the older child and adolescent
Clinical Endocrine Biochemist*
Facilitate timely analysis of samples
Provide specialist support and interpretation of results
Guide subsequent biochemical tests
Facilitate storage of samples for analysis at a later stage
Clinical Geneticist*
Facilitate timely analysis of karyotype
Closer involvement in the child with dysmorphic features
Oversee the process of genetic analysis
Facilitate storage of samples for analysis at a later stage
Genetic counselling
Gynaecologist*
Availability at an early stage to discuss future outcome
Discuss issues related to sexual function, reproductive function and surgery
Assess the understanding and review the diagnosis
Assess the need for psychology support in the adolescent girl
Initiate and monitor long-term sex steroid therapy
Adult Endocrinologist
Investigate and manage the adolescent presenting for the first time after the age of 16 years
Liaise with other members of the MDT
Act as the link between the patient and MDT
Initiate and monitor long-term medical therapy such as steroid or sex steroid therapy
Act as the transition link for adolescents under paediatric care

MDT, multidisciplinary team.

## Psychological support for the affected person and family

Early psychological input, provided by a specialist clinical psychologist with experience of supporting people with DSD and their parents, will allow the latter to examine and understand their early emotional reactions as well as explore present and future worries, adjust to the period of uncertainty during the diagnosis process and facilitate inclusion in informed decision making about themselves or their child.^6^,^7^ The clinical psychologist is also well placed to assess how well the family is coping and functioning, assess and facilitate the bonding of the parents with the infant, and, in the case of the young person, perform an assessment of gender identity, when appropriate. As a minimum, the parents of every newborn with suspected DSD where there has been a delay in sex assignment should be offered clinical psychology input. In addition, all adolescents with a newly diagnosed DSD or existing DSD requiring medical or surgical attention should be routinely offered clinical psychology input in addition to any support offered to their parents or wider family. The point of transfer from paediatric to adult services offers an ideal opportunity for a routine assessment of the need for clinical psychology input.

Discussions with parents and young people need to occur on multiple occasions in a quiet and peaceful setting, with enough time for the family and MDT to develop a shared understanding of investigations, results, diagnosis, treatments and the value of ongoing psychological support for both themselves and/or their child. The pace of how information is shared should be set by the family, and issues of confidentiality discussed and respected.^8^ Parents' and young people's initial recollections of conversations with professionals may have a long-lasting effect on them and their relationship with their affected child and health professionals.^9^ The use of phrases such as ‘differences’ or ‘variations’ in sex development may help to introduce the concept of the range of variation that may occur in sex development. A record of early discussions, either as audiotapes or a letter, which is shared between the parents and other immediate members of the MDT and the general practitioner may be helpful. Use of drawings and written material during discussion and a list of websites and support groups are useful aids for families.

Parents and young people need to be aware that the management of the condition will require a stepwise approach that first targets short-term goals and then long-term goals that achieve optimal long-term well-being. It is very likely that families' decisions will be shaped by their own expectations, experiences and their understanding of sex and gender roles within the religious and cultural context of their own social networks. The MDT should also be aware of how their own values and beliefs are played out in consultation with the family. Some parents may consider early genital surgery as a mechanism that could possibly protect their child from the risk of future stigma. This will require a thorough discussion with several members of the MDT team including the clinical psychologist, surgeons, gynaecologist and nurses so that the parents are fully informed around the controversies around undertaking or withholding early genital surgery.^10^ Parents and young people will need support and guidance about how to share news following the birth of their child/diagnosis and manage the social challenges they may face at this time.^8^,^11^

## The role of the support group

Support groups can provide ongoing support to parents and the affected individual, including opportunities to gather and explore information, promote autonomy and build knowledge and self-confidence regarding the diagnosis of DSD. For parents, gathering, using and questioning information will shape their understanding as they often act as the advocate for their child or young person and therefore need to be fully informed about DSD practice, short- and long-term outcomes of treatments and health risks and psychological challenges for their child. Support groups can provide a range of such information via websites and newsletters as well as through phone helplines and group meetings for both families and professionals.^9^ They can also work in collaboration with the MDT to help families as well as affected people in seeking appropriate medical care and improve patients' understanding of their condition as well as the reasons for medical therapy.^6^ Alongside the formal psychological support provided by the specialist clinical psychologist, support groups can also offer invaluable peer support to families and individuals affected by DSD. By being in touch with others with a similar condition and belonging to a support group, people can gain a sense of empowerment and the whole experience may also normalize a condition which may have previously been perceived as a source of stigma and shame.^12^ Healthcare professionals rely on support groups for guidance on the development of healthcare strategy as well as for providing the opportunity to interact with affected people at national support group meetings and conferences.^13^ Contact details of support groups should be supplied as routine as part of any written information. It is possible that families may prefer to talk to other local families affected in a similar way and regional services should attempt to create a local pool of helpers and explore locally organized support and education days.

## Which newborn should be investigated and how extensively?

If the appearance of the external genitalia is sufficiently ambiguous to render sex assignment impossible or the phenotype is not consistent with prenatal genetic tests, then *de facto*, extensive investigation is required. However, the extent of genital ambiguity may depend on the expertise of the observer, and prior to presentation to a clinical expert, the label of ambiguous genitalia is often assigned to newborns where the most appropriate sex of rearing is not immediately clear to those present at the child's birth. The birth prevalence of genital anomalies may be as high as one in 300 births^14^ but the birth prevalence of complex anomalies that may lead to true genital ambiguity on expert examination may be as low as one in 5000 births.^15^

**Fig. 1:**
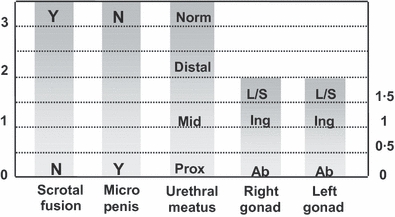
Calculating the External Masculinisation Score provides an objective aggregate score of the extent of masculinization of the external genitalia. Each individual feature of the genitalia (phallus size, labioscrotal fusion, site of the gonads and location of urethral meatus) can be individually scored to provide a score out of 12. ^1^Microphallus refers to a phallus below the male reference range. ^2^L/S, labioscrotal; Ing, inguinal; Ab, abdominal or absent on examination.

Besides those whose genitalia are truly ambiguous, in the clinical situation, infants can often be divided into those who are apparently a boy with atypical genitalia and those who are apparently a girl with atypical genitalia. However, it is very important to bear in mind that the same girl with congenital adrenal hyperplasia (CAH) may present as an apparent girl with clitoromegaly or an apparent boy with bilateral undescended testes. When evaluating these infants, the clinical features of the external genitalia that require examination include the presence of gonads in the labioscrotal folds, the fusion of the labioscrotal folds, the size of the phallus and the site of the urinary meatus on the phallus, although the real site of the urinary meatus may, sometimes, only become clear on surgical exploration.^16^ These external features can be individually scored to provide an aggregate score, the external masculinization score (EMS; Fig. 1).^17^ Routine systematic examination of 423 consecutive, apparently healthy, term newborn boys revealed that 412 (98%) had the maximum EMS of 12, 10 had an EMS of 11 and only 1 of 423 had an EMS of <11.^17^ In boys with genital anomalies, a chromosomal anomaly may be present in approximately 3% of those with isolated cryptorchidism, 7% of those with hypospadias and 13% of those with a combination of cryptorchidism and hypospadias.^18^ In infants with proximal hypospadias (penoscrotal, scrotal, perineal), detailed biochemical and molecular studies performed a decade ago revealed a likely cause in 31% of cases.^19^ With advances in biochemical and molecular techniques, it is unclear whether the diagnostic yield is even greater now.

Infants with suspected DSD who require further clinical evaluation and need to be considered for investigation by a specialist should include those with isolated perineal hypospadias, isolated micropenis, isolated clitoromegaly, any form of familial hypospadias and those who have a combination of genital anomalies with an EMS of <11. This will avoid unnecessary detailed investigations into boys with isolated glandular or mid-shaft hypospadias and boys with unilateral inguinal testis. The co-existence of a systemic metabolic disorder, associated malformations or dysmorphic features would lower the threshold for investigation as would a family history of consanguinity, stillbirths, multiple miscarriages, fertility problems, genital abnormalities, hernias, delayed puberty, genital surgery, unexplained deaths and the need for steroid replacement. In addition, maternal health and drug exposure during pregnancy and the pregnancy history itself may hold key information.

In all infants with ambiguous genitalia and/or bilateral impalpable gonads, a first tier of investigations should be undertaken to define the sex chromosomes and delineate, by pelvic ultrasound, the internal genitalia and exclude life-threatening CAH – the commonest cause of ambiguous genitalia of the newborn. This first tier should, therefore, also include plasma glucose, serum 17OH-progesterone (17OHP) and serum electrolytes. Serum 17OHP is usually unreliable before the age of 36 h, and in the salt-losing form of CAH, serum electrolytes usually do not become abnormal before day 4 of life. The results of PCR or FISH analysis using Y and X-specific markers should be available within one working day and the 17OHP results should be available with a maximum of two working days in all specialist DSD centres. In situations where the level of suspicion of CAH is very high and the infant needs immediate steroid replacement therapy, further serum samples should be collected and stored before starting therapy. These should be of a sufficient volume to assess 17OHP, testosterone, androstenedione and, possibly, renin activity or concentration, in that order of priority. At least one spot or 24-h urine sample (at least 5 ml) for a urine steroid profile should be collected before starting therapy. The results of these initial investigations shall often dictate the second tier of investigations.

In an infant with impalpable gonads, a karyotype of 46,XX, a significantly elevated serum 17OHP and the presence of a uterus make CAH because of 21-hydroxylase deficiency very likely. A urine steroid profile can confirm this diagnosis and can also identify other rare forms of CAH that may also be associated with a raised 17OHP in the newborn. In infants with sex chromosomes other than 46,XX, a second tier of investigations is necessary to determine the presence of testes and the adequacy of androgen production and action. These tests include measurement of serum anti-Müllerian hormone (AMH), the human chorionic gonadotrophin (hCG) stimulation test, further detailed imaging and laparoscopy. Confirmation of a specific diagnosis will often require further biochemical identification of a defect in the androgen biosynthesis pathway and detailed genetic analysis.

## Which adolescent should be investigated and how extensively?

The initial assessment in an affected adolescent should be aimed at establishing a relationship with the patient and starting the process of diagnosis. In adolescents with an existing DSD, transferring to adult services is an opportunity to review the diagnosis and consider further investigations. An appropriate hospital setting is very important for the sensitive management of complex conditions with full access to the necessary medical, nursing and psychological care. Whilst the explanation of the diagnosis to the patient and the family is critical, this needs to be performed sensitively and carefully and expert psychological input is essential.

**Fig. 2:**
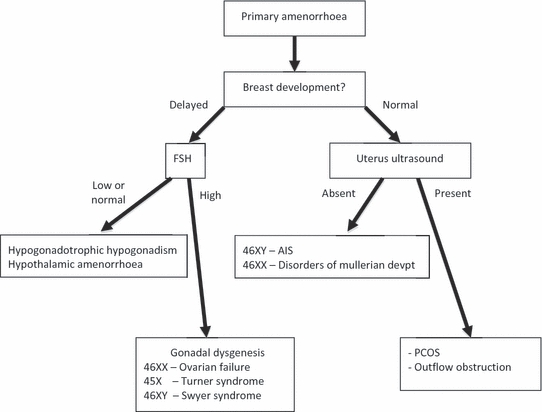
Approach to investigating adolescent girls with primary amenorrhoea.

Adolescents may typically present with a suspected DSD in three ways – a girl with primary amenorrhoea (with or without breast development), a girl who virilizes at puberty or a boy with pubertal delay (Fig. 2). The potential psychological impact of examinations and medical photography should be considered, and for the adolescent, a thorough physical examination by a surgeon and a gynaecologist, depending on the sex of the adolescent, may only be appropriate under an anaesthetic.^20^

In girls with primary amenorrhoea, investigations should be considered at the age of 14 years if there is no pubertal development and at 16 years if other aspects of puberty, particularly breast development, have progressed normally. History should include a family history and an assessment of co-existing chronic disease, exercise and weight changes. Physical examination should include measurement of blood pressure, height and weight and assessment of secondary sexual characteristics including clitoral enlargement. Vaginal examination to assess vaginal length is rarely indicated when imaging is informative and should be clearly explained and performed by a gynaecologist. An initial investigation screen should comprise measurements of serum electrolytes, LH, FSH, prolactin, TSH, FT4, sex hormone binding globulin (SHBG), androstenedione, oestradiol, testosterone and transabdominal pelvic ultrasound by sonographer who has experience of adolescent appearances. Raised gonadotrophins or an absent uterus in the presence of normal breast development are indications for a karyotype.

The appearance of clitoromegaly and hirsutism at puberty in the presence of primary amenorrhoea is a classical presentation of two 46,XY DSDs: 17β-hydroxysteroid dehydrogenase type 3 (17βHSD3) deficiency and 5α-reductase type 2 deficiency. It is less typical of partial androgen insensitivity syndrome (PAIS) which is usually associated with ambiguous genitalia at birth. In all these conditions, Müllerian structures will not be detectable. Also, in partial gonadal dysgenesis and ovotesticular DSD, the mild clitoromegaly that may have been present at birth may have been overlooked but becomes a more prominent feature at adolescence. The differential diagnosis would also include CAH and androgen-secreting tumours of the ovary or adrenal gland; in all these cases, Müllerian structures are present. Investigations include serum measurements of LH, FSH, dehydroepiandrosterone (DHEAS), SHBG, androstenedione, testosterone, dihydrotestosterone (DHT) and 17OHP. A 24-h urine collection for urinary steroid profile (USP) will confirm 5α-reductase type 2 deficiency, CAH or adrenocortical tumour. A pelvic ultrasound will assess the presence of a uterus and determine the need for a karyotype.

Although the commonest cause of delayed puberty is constitutional delay, all boys with delayed puberty who are over the age of 14 years should be assessed. Overweight boys need careful examination so that a buried penis is not mistaken for micropenis. Rarely, PAIS, a disorder of testosterone biosynthesis or mild forms of testicular dysgenesis, can present in this age group, especially if there is a history of hypospadias repair or orchidopexy. Investigations include a bone age and serum measurements of LH, FSH, testosterone and prolactin. For those with raised gonadotrophins, karyotype should be performed to exclude disorders such as Klinefelter's syndrome (47,XXY and variants) or 45,X/46,XY mosaicism.

## The role of the clinical geneticist

**Fig. 3:**
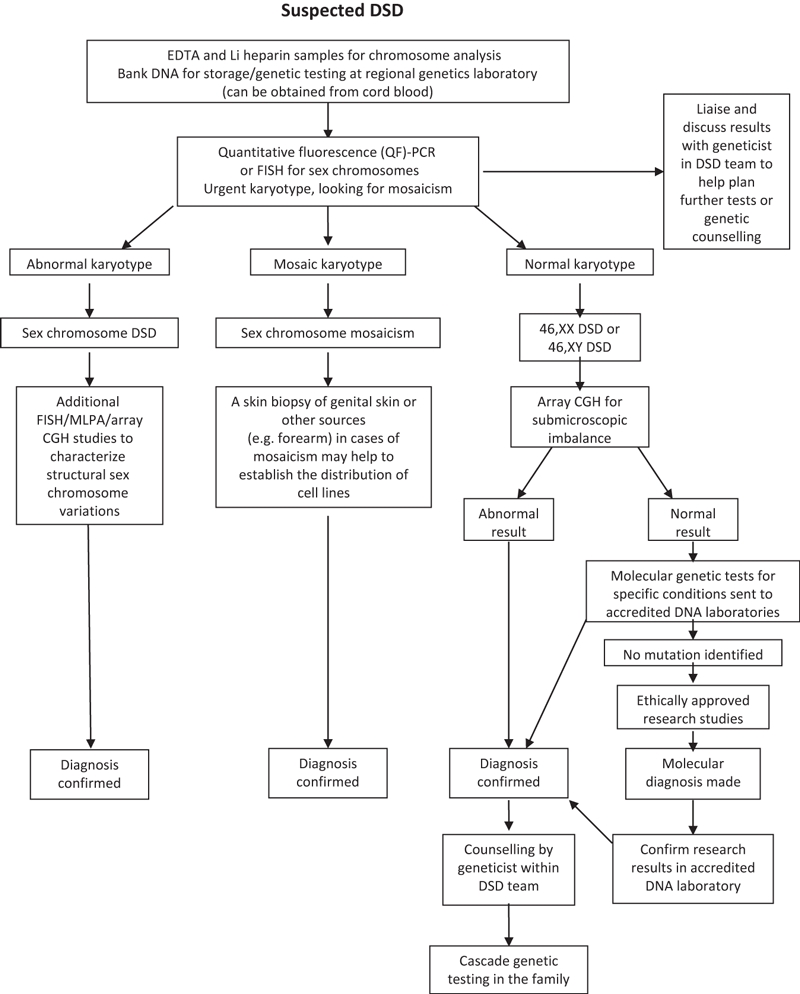
Role of the clinical genetics service within the specialist DSD team. FISH, fluorescence *in situ* hybridization; MLPA, Multiplex Ligation-dependent Probe Amplification.

Establishing a specific molecular diagnosis is helpful in clinical management of cases and in offering accurate genetic counselling for the family. However, the number of diagnostic gene tests that are available in accredited DNA laboratories in the United Kingdom or internationally is limited and costly. As recent developments in DNA and chromosomal analysis accelerate biomedical research, many techniques such as multiplex ligation-dependent probe amplification (MLPA) and comparative genomic hybridization (CGH) have the potential to become routine in clinical practice. Next-generation DNA sequencing platforms will allow whole-genome sequencing for rare diseases to become a reality at a realistic price.^21^,^22^ The clinical geneticist at the specialist DSD centre is correctly placed to evaluate complex genetic syndromes and also judge which technique is appropriate and cost-effective for each clinical situation. DNA can be stored indefinitely with consent, so when further diagnostic testing opportunities arise, aliquots of DNA can be accessed, avoiding repeated venepuncture. If a molecular diagnosis is reached in a research laboratory, then confirmation of this result in an accredited laboratory should be sought where possible, before disclosing results to the family. Close involvement of the clinical genetics service can ensure that the MDT covers all aspects of genetic counselling including provision of information to the family, the mode of inheritance of the disorder and the choices or options available for dealing with this risk. Established links with the clinical genetics service are also useful when considering prenatal testing or interventions such as steroid therapy in CAH (Fig. 3).

## Assessment of anatomy

Examination and assessment by a paediatric surgeon with experience of DSD is critically important in the affected newborn. Combining expert physical examination with endoscopic visualization and radiological assessment can provide information on the location and state of the gonads, the urogenital sinus and Müllerian structures. During this initial assessment, the anatomy and drainage of the renal tract should also be assessed.^23^,^24^

Ultrasonography is the first-line imaging modality and should include the adrenals, kidneys, pelvis, inguinal regions and scrotum where appropriate. In the neonate, the uterus, ovaries and adrenals should be identifiable but the reliability is child and operator dependent. It should also be borne in mind that the presence of a uterine structure does not guarantee later function, and intra-abdominal testes and streak gonads are difficult to identify ultrasonography. In the adolescent, it is sometimes difficult to confirm the presence of a prepubertal uterus by ultrasonography and there may be a place for repeat imaging after a 6-month course of oestrogen. Magnetic resonance imaging (MRI) should be reserved for cases where ultrasonography has failed to delineate the relationship of the Müllerian structures and where there are abnormalities of the urinary tract. High-resolution MRI should include the pelvis and perineum, using high-resolution T2 with and without fat saturation and T1 in three planes where possible. MRI can identify extra-abdominal ectopic testes and the presence of the spermatic cords, but is of less value in trying to define the presence and character of intra-abdominal testes or streak gonads. In adolescents, MRI can delineate structural anomalies such as hydrometrocolpos or hydronephrosis and identify secretory tumours. In general, T1 imaging of the upper abdomen in adolescents is not required unless there is an adrenal mass in which case contrast enhancement shall also be required.^25^,^26^

Nowadays, the ‘genitogram’ is not routinely performed for diagnostic purposes. It has been superseded by endoscopic examination of the genital tract (genitoscopy), which provides a more detailed and thorough assessment. However, at the time of surgery, stents can be accurately placed in various structures to allow a more focused radiological examination. These investigations need to provide information on the length of the urogenital sinus, the associated Müllerian structures and the relationship of the urethra and its sphincter. In 46,XX DSD, genitoscopy can assess the need for drainage of both the bladder and Müllerian structures and provide a detailed assessment of the urogenital anatomy. In 46,XY DSD, endoscopic examination can be used to identify any Müllerian remnants that arise from the posterior urethra.

Genitoscopy can be augmented by laparoscopy, but this is not necessary in all cases of DSD. It is a very effective method of visualizing the internal sex organs and facilitates direct inspection, biopsy or excision of intra-abdominal gonads. However, as laparoscopy can only visualize intraperitoneal structures, Müllerian remnants deep within the pelvis or closely attached to the bladder may not be seen. In 46,XY DSD, laparoscopy is clearly indicated in all infants with impalpable testes where the gonads need to be identified and brought down to the scrotum if possible. Laparoscopy can also be used in adolescents who present with a DSD. However, MRI may be a more suitable first-line investigation for defining the anatomy.

## Steroid measurement and its interpretation

Steroid hormone analysis is a vital component of the biochemical evaluation but the method of analysis can have a significant impact on the result.^27^ Analysis is most often performed by nonextraction, nonchromatographic (direct) immunoassays on automated platforms and these are subject to concerns of analytical specificity.^28^,^29^ Liquid chromatography linked with tandem mass spectrometry (LC-MS/MS) allows multiple analyte analysis from a single sample whilst maintaining analytical specificity.^30^ Thus, in cases of DSD, plasma or serum steroids should be measured by either LC-MS/MS or immunoassays after organic solvent extraction. As these are more labour intensive, there may be an impact on the turnaround time for results and on clinical decision making. Close communication between the clinical and biochemistry personnel within the DSD team is vital to enable correct interpretation of laboratory results and awareness that results should be available in a timely manner.

Urinary steroid profile analysis by gas chromatography mass spectrometry (GC-MS) provides qualitative and quantitative data on excretion of steroid metabolites. It is ideal for detecting altered steroid metabolites, especially in cases of CAH where the activity of a combination of steroidogenic enzymes can produce unusual metabolites that can cross-react in traditional serum assays.^31^ The diagnosis of rarer forms of CAH such as P450 oxidoreductase deficiency (ORD) is best established using urinary GC-MS analysis as it allows for concurrent determination of all adrenal-derived steroid metabolites.^32^ As gonadotrophins, androgens and precursors, fluctuate markedly over the first few months of life and may lead to a diagnostically blind window, there is a place to consider an early neonatal collection as well as further samples at a later stage. A urine sample can be frozen and stored for many years and may help with a review of the diagnosis at a later stage. USP is not appropriate for suspected cases of 5α-reductase type 2 deficiency until after 3 months of age as diagnostic pairs of 5β to 5α reduced metabolites are not detectable until then.

Normally, infants, particularly boys, have significant changes in steroid and other endocrine hormone concentrations during the first 100 days of birth.^28^,^33^ In boys, serum testosterone and DHT may initially be high at birth but decline to <1 nmol/l or undetectable, respectively. Concentrations then rise from around day 30 after birth to peak at day 70 before declining to normal prepubertal concentrations.^33^ These normal variations may influence the interpretation of sex steroid and gonadotrophin measurements as well as the results of the hCG stimulation test. Furthermore, the actual value for the hormone concentration will vary depending on the assay methodology.

## Serum Anti-Müllerian hormone

Anti-Müllerian hormone, also known as Müllerian-inhibiting substance (MIS), is strongly expressed in Sertoli cells from the time of testicular differentiation to puberty and to a much lesser degree in granulosa cells from birth to menopause and is widely used nowadays to assess ovarian reserve.^34^ Published information on circulating AMH concentrations have to be interpreted with caution because of differences in the way immunoassays are standardized and the units used for measurement. It is, therefore, important to liaise with the specialist clinical biochemist to ensure appropriate reference ranges are used for interpretation. In boys, AMH is detectable at birth at much higher circulating concentrations than in girls and these concentrations rise over infancy before gradually declining at puberty. Therefore, up to date, age-, sex- and method-related reference ranges are necessary for interpretation.^35^ In male neonates, levels that are close to the lower end of the normal range should be repeated later in infancy as they should rise further in boys with normal testes. As summarized in Table 2, measurement of AMH is a powerful tool to assess Sertoli cell activity in children with suspected DSD and may also have a diagnostic utility in conditions associated with androgen deficiency or insensitivity (Table 2).^36^

**Table 2:** Interpretation of serum AMH concentration in DSD

Serum AMH	Testicular tissue	Interpretation
Undetectable	Absent	46,XX CAH
		Complete gonadal dysgenesis
		PMDS due to AMH gene defect
Within female age-related reference range	Usually absent	46,XX CAH
		Dysgenetic testes or ovotestes
Below male or above female age-related reference range	Present	Dysgenic testes
		Ovotestes
Within male age-related reference range	Usually normal	Nonspecific XY DSD
		Hypogonadotrophic hypogonadism
		PMDS due to AMH-R defect
		46,XX testicular DSD
		Ovotestes
Above male age-related reference range	Present	AIS especially complete androgen insensitivity syndrome 5α-reductase deficiency
		Testosterone biosynthetic defect
		Leydig cell hypoplasia

AMH, anti-Müllerian hormone; CAH, congenital adrenal hyperplasia; DSD, Disorders of sex development.

## The human chorionic gonadotrophin stimulation test

**Fig. 4:**
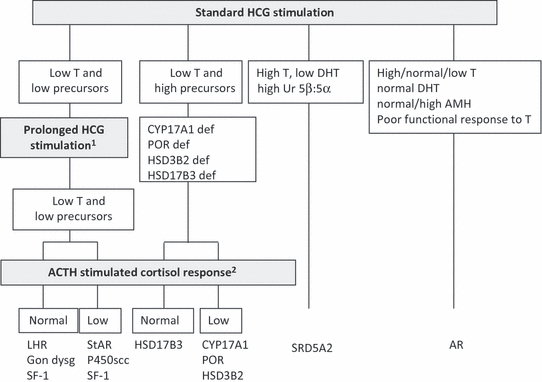
Interpretation of the results of the human chorionic gonadotrophin (hCG) stimulation test when investigating XY DSD and pointers for consideration of prolonged hCG stimulation and adrenocorticotrophin hormone stimulation. ^1^Prolonged hCG stimulation test should be considered in those cases where there is a poor testosterone (T) response to a standard hCG stimulation test. ^2^Synacthen stimulation test should be considered in those cases who show a poor testosterone response to hCG stimulation. 46,XY children with lipoid congenital adrenal hyperplasia due to a steroidogenic acute regulatory defect or P450scc deficiency due to CYP11A1 defect will have female genitalia and present in a salt-losing state in the first days or weeks of life before synacthen performed.

Stimulation with hCG allows the identification of functioning testicular tissue as well as biosynthetic defects in testosterone synthesis (Fig. 4). However, it is an invasive test that should only be performed as a second-line investigation after discussion with the paediatric endocrinologist in the regional DSD team. Most protocols for hCG stimulation in the United Kingdom use intramuscular hCG 1000–1500 units on three consecutive days.^37^ This can be followed by further hCG stimulation with 1500 units on 2 days a week for the following 2 weeks. In young infants and adolescents, 3 days of hCG stimulation may be sufficient,^38^ and in the very young infant with an intrinsically active gonadal axis, an hCG stimulation test may not be necessary if serial blood samples show raised serum testosterone concentrations. A testosterone response to hCG may be labelled as normal if absolute testosterone concentrations reach a level that is above the upper limit of the normal prepubertal range or rise by more than twice the baseline value. As a minimum, other androgens that should be assessed include DHT and androstenedione. For these two metabolites, the post-hCG, day 4 sample is more important than the pre-hCG, day 1 sample and there does not seem to be any additional benefit of analysing a sample for these two metabolites on day 22, following prolonged hCG stimulation, if there are sufficient samples to analyse on day 4.^38^ The day 22 sample that is collected for testosterone measurement should be stored and can be used to measure DHT or androstenedione if a sufficient sample was not available at day 4.

In routine cases of XY DSD, a persistently low AMH may have a high predictive value for a low hCG-stimulated testosterone concentration but a normal AMH has a low predictive value for a normal hCG-stimulated testosterone value.^35^ These relationships between the two variables do not apply to cases of persistent Müllerian duct syndrome where there is an intrinsic defect of AMH or the AMH receptor. There is no evidence that a urine steroid profile or a serum AMH checked after hCG stimulation has any added diagnostic value. In the presence of a poor testosterone response following hCG stimulation, assessment of adrenal function by a standard short synacthen stimulation test should be considered in all cases. There is less experience as well as a lower demand for a corresponding test to assess ovarian tissue but recent reports of ovarian hormones following stimulation with FSH need to be explored further.^39^,^40^ There is currently insufficient evidence to recommend that everybody with XY DSD should have a synacthen stimulation test but clinicians should be aware of the clear association between some forms of DSD and primary adrenal insufficiency and should consider thorough assessment of adrenal function in those diagnoses where an association has already been described and in those with any clinical suspicion of adrenal insufficiency, especially those with low steroid precursors on USP.

## XX DSD

46,XX DSD can be classified into disorders of ovarian development, conditions with androgen excess and other syndromes, which are often associated with other developmental abnormalities.

Congenital adrenal hyperplasia is the commonest cause of 46,XX DSD with ambiguous genitalia in the neonatal period or early infancy and is characterized by androgen excess and a variable alteration in glucocorticoid and mineralocorticoid function and a specific profile of steroid hormones.^41^,^42^ This profile can identify the enzyme defects including deficiency of 21α-hydroxylase (90–95% of cases), 11β-hydroxylase (4–8% of cases), 3β-hydroxysteroid dehydrogenase type 2 (3βHSD2; rare) and P450 oxidoreductase (unknown prevalence). P450 ORD biochemically manifests as apparent combined CYP17A1 and CYP21A2 deficiency, sometimes also resembling CYP19A1 (aromatase) deficiency. Unlike other forms of CAH, ORD is characterized by increased androgen concentrations only during the prenatal and early neonatal period, but rapidly develop sex hormone deficiency. Further details of these enzyme defects are outlined in Table 3.

**Table 3:** Characteristics of 46, XX disorders of sex development due to androgen excess

	Inheritance and Gene	Genitalia	Wolffian duct derivatives	Müllerian duct derivatives	Gonads	Typical signs and symptoms	Hormone profile
21-hydroxylase def	Autosomal Recessive, *CYP21A2*	Ambiguous	Absent	Normal	Ovary	Severe adrenal insufficiency in infancy ± salt loss; moderate to severe androgenization at birth	Decreased cortisol and/or mineralocorticoids.
							Increased 17-hydroxyprogesterone, 21-deoxycortisol, androstenedione, testosterone, and/or plasma renin (activity)
11β-hydroxylase def	Autosomal Recessive, *CYP11B1*	Ambiguous	Absent	Normal	Ovary	Adrenal insufficiency in infancy; moderate to severe androgenization at birth; arterial hypertension often developing at different ages	Decreased cortisol, corticosterone, aldosterone, and/or plasma renin (activity)
							Increased 11-deoxycortisol, 11-deoxycorticosterone, androstenedione, testosterone
3β-hydroxysteroid dehydrogenase II def	Autosomal Recessive, *HSD3B2*	Commonly clitoromegaly or mild virilization, also normal	Absent	Normal	Ovary	Severe adrenal insufficiency in infancy ± salt loss, androgenization during childhood and puberty, premature pubarche	Increased concentrations of Δ^5^ C_21_- and C_19_- steroids, 17 hydroxypregnenolone and DHEA suppressible by dexamethasone
P450 oxidoreductase def	Autosomal Recessive, *POR*	Ambiguous or normal female	Absent	Normal	Ovary	Variable androgenization at birth and puberty, glucocorticoid deficiency, features of skeletal malformations.	Combined P450c17 and P450c21 insufficient, normal or low cortisol with poor response to ACTH stimulation, elevated 17-hydroxyprogesterone, testosterone, progesterone and corticosterone; low oestradiol.
						Maternal androgenization during pregnancy onset second trimester possible	
P450 aromatase def	Autosomal Recessive, *CYP19A1*	Ambiguous	Absent	Normal	Ovary	Delayed bone age, development of ovarian cysts during infancy, childhood and puberty. Maternal androgenization during pregnancy	High androgens in cord blood, androgens may stay elevated or normalize soon after birth

46,XX DSD also includes disorders of gonadal development including 46,XX ovotesticular DSD and 46,XX testicular DSD. 46,XX ovotesticular DSD commonly presents at birth with ambiguous genitalia and progressive virilization during puberty. In contrast, individuals with 46,XX testicular DSD usually have a normal male phenotype and absent Müllerian structures and are often diagnosed after karyotype analysis during work-up for infertility. In 46,XX testicular DSD, about 80–90% of patients will have Y chromosomal material including a translocated *SRY* gene, which is only rarely detected in 46,XX ovotesticular DSD. In other cases of 46,XX testicular DSD, duplication or upregulation of the *SOX9 or SOX3* gene and mutations of the *RSPO1* gene have been described. In those with a suspicion of 46,XX ovotesticular DSD, functional testing will require detection of testicular and ovarian tissue by a combination of biochemical testing, imaging and surgical exploration.

Disorders of Müllerian development are another group of 46,XX DSD, and in these cases, ovarian function is usually normal but often associated with cloacal anomalies and other characteristic malformations. Although most cases of Müllerian development disorders are not associated with androgen excess, the presence of the latter, particularly in the adolescent, should alert the clinician to a possible abnormality of the *WNT4* gene.

## XY DSD with low testosterone and low precursors

The differential diagnosis of 46,XY DSD associated with low testosterone and low precursors includes the following: high defects in steroid synthesis (steroidogenic acute regulatory (StAR) protein, P450side chain cleavage(scc) enzyme/*CYP11A1*, sometimes Smith-Lemli-Optiz/*DHCR7*); LH receptor defects (*LHCGR*); and partial and complete forms of gonadal (testicular) dysgenesis (Table 4). Of note, complete or partial combined 17α-hydroxylase/17,20-lyase deficiency (*CYP17A1*) may also present with ‘low testosterone and low precursors’ if DHEAS and androstenedione are the only intermediates measured. The actual diagnosis can be reached by assessment of adrenal function by measuring ACTH, ACTH-stimulated cortisol, PRA, DOC, corticosterone, aldosterone, measurement of Δ5 (pregnenolone, 17OHPreg) and Δ4 (progesterone, 17OHP) precursors or urine steroid analysis. Isolated 17,20-lyase deficiency and ORD might also be diagnosed by this approach. Proximal blocks (StAR, P450scc) in the pathway affect steroidogenesis in the adrenal gland as well as the developing gonad.

**Table 4:** Characteristics of 46, XY disorders of sex development

	Inheritance and Gene	Genitalia	Wolffian duct derivatives	Mullerian duct derivatives	Gonads	Typical features	Hormone profile
Leydig cell hypoplasia	Autosomal Recessive, *LH/HCGR*	Female, hypospadias or micropenis	Hypoplastic	Absent	Testes	Under androgenization with variable failure of sex hormone production at puberty	Low T and DHT, elevated LH and FSH, exaggerated LH response to LHRH, poor T and DHT response to hCG stimulation
Lipoid CAH	Autosomal Recessive, *StAR*	Female, rarely ambiguous or male	Hypoplastic or normal	Absent	Testes	Severe adrenal insufficiency in infancy with salt loss, failure of pubertal development, rare cases associated with isolated glucocorticoid deficiency	Usually deficient of glucocorticoids, mineralocorticoids and sex steroids
P450SCC def	Autosomal Recessive, *CYP11A1*	Female, rarely ambiguous or hypospadias	Hypoplastic or normal	Absent	Testes	Severe adrenal insufficiency in infancy with salt loss ranging to milder adrenal insufficiency with onset in childhood	Usually deficient of glucocorticoids, mineralocorticoids and sex steroids
3β-hydroxysteroid dehydrogenase II def	Autosomal Recessive, *HSD3B2*	Ambiguous, hypospadias	Normal	Absent	Testes	Severe adrenal insufficiency in infancy ± salt loss, poor androgenization at puberty with gynaecomastia	Increased concentrations of Δ^5^ C_21_- and C_19_- steroids, 17 hydroxypregnenolone and DHEA suppressible by dexamethasone
Combined 17α-hydroxylase/17,20-lyase def	Autosomal Recessive, *CYP17A1*	Female, ambiguous, hypospadias or micropenis	Absent or hypoplastic	Absent	Testes	Absent or poor virilization at puberty, gynaecomastia, hypertension	Decreased T, increased LH and FSH, increased plasma deoxycorticosterone, corticosterone and progesterone, decreased plasma renin activity, low renin hypertension with hypokalaemic alkalosis
Isolated 17,20-lyase def	Autosomal Recessive, *CYP17A1*, usually affecting key redox domains, alternatively caused by cytochrome b5 mutations (*CYB5*)	Female, ambiguous or hypospadias	Absent or hypoplastic	Absent	Testes	Absent or poor androgenization at puberty, gynaecomastia	Decreased T, DHEA, androstenedione and oestradiol, abnormal increase in plasma 17-hydroxyprogesterone and 17-hydroxypregnenolone, increased LH and FSH, increased ratio of C_21_-deoxysteroids to C_19_-steroids after hCG stim
P450 oxidoreductase def	Autosomal Recessive, *POR*	Ambiguous, hypospadias or normal male	Absent or hypoplastic	Absent	Testes	Variable androgenization at birth and puberty, glucocorticoid deficiency, features of skeletal malformations.	Combined P450c17 and P450c21 insuff, normal or low cortisol with poor response to ACTH stim, elevated 17-hydroxyprogesterone, T low
						Maternal androgenization during pregnancy onset second trimester possible	
17β-hydroxysteroid dehydrogenase type 3 def	Autosomal Recessive *HSD17B3*	Female, ambiguous, blind vaginal pouch	Present	Absent	Testes	Androgenisation at puberty, gynaecomastia variable	Increased plasma estrone, decreased ratio of testosterone/androstenedione and oestradiol after hCG stim, increased FSH and LH
5α-reductase-2 def	Autosomal Recessive *SRD5A2*	Ambiguous, micropenis, hypospadias, blind vaginal pouch	Normal	Absent	Testes	Decreased facial and body hair, no temporal hair recession, prostate not palpable	Decreased ratio of 5α/5β C_21-_ and C_19-_ steroids in urine, increased T/DHT ratio before and after hCG stim, modest increase in LH, decreased conversion of T to DHT *in vitro*
CAIS	X-linked recessive*AR*	Female with blind vaginal pouch	Often present depending on mutation type	Absent or vestigial	Testes	Scant or absent pubic and axillary hair, breast development and female body habitus at puberty, primary amenorrhoea	Increased LH and T, increased oestradiol, FSH levels normal or slightly increased, resistance to androgenic and metabolic effects of T (may be normal in some cases)
PAIS	X-linked recessive *AR*	Ambiguous with blind vaginal pouch, isolated hypospadias, normal male with infertility (mild)	Often normal	Absent	Testes	Decreased to normal axillary and pubic hair, beard growth and body hair, gynaecomastia common at puberty	Increased LH and T, increased oestradiol, FSH levels may be normal or slightly increased, partial resistance to androgenic and metabolic effects of T

DHT, dihydrotestosterone; FSH, follicle-stimulating hormone; hCG, human chorionic gonadotrophin; LH, luteinizing hormone; T, testosterone; DHEA, dehydroepiandrosterone; ACTH, adrenocorticotrophin hormone; AR, androgen receptor; CAIS, complete androgen insensitivity syndrome; PAIS, partial androgen insensitivity syndrome.

Luteinizing hormone receptor defects (‘Leydig cell hypoplasia’) typically result in elevated basal LH, hyperresponsive LH to GnRH stimulation, low precursors and testosterone, and impaired androgen response to hCG stimulation. No Müllerian structures will be present and adrenal function is normal. A spectrum of phenotypes has been reported including ambiguous genitalia and micropenis. In some cases, basal LH may not be elevated at times when the HPG axis is quiescent (6 months to late childhood).

In complete gonadal dysgenesis (‘Swyer syndrome’), affected people will usually have a female phenotype with intra-abdominal streak gonads. In some situations, ovotestes or even undifferentiated gonadal tissue may be found.^43^–^45^ Müllerian structures are usually present because of impaired AMH secretion in early foetal life. Androgens and their precursors will be low, LH elevated, depending on age, and a poor or absent testosterone response to hCG stimulation is seen. AMH concentrations will be low or undetectable and adrenal function is usually normal unless the underlying defect is in steroidogenic factor-1 (*SF-1*) or related adrenal or gonadal factors.

Partial gonadal (testicular) dysgenesis can present with a spectrum of phenotypes ranging from clitoromegaly, to ambiguous genitalia or severe hypospadias. Müllerian structures may or may not be present, and testes of variable size and architecture are present along the path of descent. The biochemical profile is similar to complete gonadal dysgenesis, but generally less severe. If mild degrees of clitoromegaly in infancy are overlooked, a 46,XY child with partial gonadal dysgenesis may first present at puberty with progressive androgenization. Genetic analysis and associated features may be useful in defining the molecular aetiology of some forms of gonadal dysgenesis.^46^

## XY DSD with low testosterone and high steroid precursors

46,XY DSD with low testosterone and increased precursors can be caused by several variants of CAH, namely by 17α-hydroxylase (CYP17A1) deficiency, ORD and 3βHSD2 deficiency, caused by inactivating mutations in the corresponding genes *CYP17A1*, *POR* and *HSD3B2*, respectively. In addition, 46,XY DSD with low testosterone and increased precursors can typically be found in individuals affected by 17βHSD3 deficiency, caused by *HSD17B3* mutations (Table 4).

Deficiency of CYP17A1 leads to CAH in about 1% of cases of 46,XY DSD. Characteristically, affected individuals present with female genitalia and low DHEA, androstenedione and testosterone. There is an increase in mineralocorticoid synthesis, and although there may be cortisol deficiency, this is rarely manifested, as corticosterone can also bind and activate the glucocorticoid receptor. In ORD, sex steroids are characteristically low, sometimes low normal, whilst pregnenolone and progesterone and their metabolites accumulate, as expression of the combined block of CYP21A2 and CYP17A1 activities. Although there is often a relative preponderance of mineralocorticoid over glucocorticoid metabolites in affected cases, hypertension only manifests in adolescence or later. Although baseline glucocorticoid secretion is usually sufficient, in the majority of cases, the stress response to ACTH is significantly impaired, requiring at least stress dose hydrocortisone cover or permanent glucocorticoid replacement. 3β-HSD2 (also termed Δ4-Δ5 isomerase) deficiency invariably leads to glucocorticoid deficiency and as well as a variable degree of mineralocorticoid deficiency and its characteristic features are outlined in Table 4. 17β-HSD3 deficiency is responsible for the conversion of androstenedione to testosterone in the gonad and has no effect on adrenal steroidogenesis. Plasma steroids characteristically show increased androstenedione levels whilst testosterone levels are concurrently low, particularly after hCG stimulation. However, a low testosterone to androstenedione ratio may also occur in cases of gonadal dysgenesis and the reliability of a low ratio in identifying 17β-HSD3 deficiency is unclear. In urine, the typical finding is an increase in the androgen (and androstenedione) metabolites, androsterone (An) and etiocholanolone (Et), but it is unclear whether this applies across all age groups.^31^

## XY DSD with normal testosterone, normal precursors and low DHT

The type 2 isoenzyme of 5α-reductase type 2 (SRD5A2) is highly expressed in androgen-sensitive tissues^47^ and converts testosterone to the more potent androgen, DHT, required for the development of external male genitalia. At birth, the external appearance of the genitalia of an infant with SRD5A2 deficiency can range from a completely female phenotype to a range of hypospadias severity or just isolated micropenis. A positive family history is often present in this autosomal recessive condition. In serum, the testosterone/DHT ratio following hCG stimulation usually exceeds 30:1. In infants over 3–6 months, the defect should be easily identifiable simply on a urine sample which shows a decreased ratio of 5α:5β-reduced C_21_ and C_19_ steroids and thus can be reached in a child who had early gonadectomy. Early diagnosis of this condition is important as the affected infant may need sex reassignment if initially raised as a girl. In the infant raised as a boy, application of topical DHT cream may be a method of assessing the potential of the genitalia to virilize over the longer term.

## XY DSD with normal testosterone, normal precursors and normal DHT

A defect in androgen signalling is most likely due to dysfunction of the androgen receptor (AR) and mutations resulting in a complete lack of function of the *AR* cause complete androgen insensitivity syndrome (CAIS).^48^ This presents in the newborn infant as a discordance between a female phenotype and a prenatal karyotype of 46,XY, a postnatal check because of a positive family history, or as inguinal swellings in an otherwise normal girl. CAIS usually presents in adolescence as primary amenorrhoea with normal breast development. The presence of pubic hair is often reported in CAIS and should not be used to exclude the diagnosis. Mutations that result in some residual AR function and varying degrees of androgenization cause PAIS. Although children with AIS typically have normal testosterone and DHT response to hCG stimulation and a normal USP, some demonstrate a poor response to hCG stimulation.^37^,^49^ The serum AMH concentration is normal or may even be elevated. LH levels are increased in the face of normal or elevated serum testosterone, reflecting a state of androgen resistance.^36^ A family history of X-linked inheritance is informative although one-third of cases are the result of spontaneous new mutations.

A functional assessment of androgen sensitivity may include assessing the clinical effect of a short course of testosterone applied on the phallus or by the effect of systemic testosterone following hCG stimulation. However, there is no consensus on the choice of androgen, dosage, method of administration, timing, duration of treatment and the definition of a satisfactory response in the growth of the phallus. Androgen sensitivity can be also assessed by measuring change in SHBG, an androgen-responsive protein which normally decreases following androgen exposure. This fall in SHBG is absent in CAIS, variable in PAIS^50^ and difficult to interpret in young infants who have highly variable circulating SHBG. *AR* analysis may reveal a mutation in more than 80% of cases with a CAIS phenotype and 30% of cases with a PAIS phenotype,^51^ and AR binding studies are not necessary for routine diagnosis of AIS. A number of cases of XY DSD are loosely labelled as ‘PAIS’ when no conclusive biochemical or genetic abnormalities are identified in gonadal function, androgen synthesis or androgen action. The term PAIS should be reserved for those children who have XY DSD and a pathogenic mutation in *AR*.

## Networks and registers for clinical care, audit and research

It is unrealistic to expect that every major clinical centre can possess a comprehensive, multidisciplinary DSD team as outlined earlier. Furthermore, in many cases, care at a local hospital may be more appropriate for reasons of both convenience and necessity (for example, adrenal crisis in CAH). For the less complex case of hypospadias, immediate multidisciplinary input may not be necessary and initial discussion and explanation of the condition with the parents does not require urgent transfer of the baby at an emotionally sensitive period. Similarly, some investigations can also be performed at local centres that are affiliated to a regional centre. It is, however, important that all personnel who may be involved in the care of an affected person have access to the regional DSD team and have the opportunity to develop themselves professionally. Some regions have overcome these hurdles with the development of a national managed clinical network (http://www.sgan.nhsscotland.com). A service model such as this ensures the provision of an equitable state-of-the-art service for all affected children and adolescents in a region. A formal organization allows a structured referral pathway within the region as well as beyond and provides the infrastructure for better long-term care of the patient as close to home as possible. A network also facilitates the creation of nationally agreed protocols for the care of the affected newborn, setting and monitoring of national standards of care, and rational utilization of other services such as clinical genetics and clinical biochemistry and provides a forum for education and professional development.

Research and audit are vital for the management of DSD, and clinical networks have a strong potential to drive these activities. The 2005 Consensus Workshop on DSD stressed the need for the creation and maintenance of a database in centres of expertise. Clinical audit systems that collect information on clinical activity and outcome should be an integral component of national specialist services. Such databases may exist at a less formal level in many other regional centres and until recently have lacked international uniformity and the ability to crosstalk. A web-based register (https://tethys.nesc.gla.ac.uk/) has been approved by the UK National Research Ethics Service as a multicentre research database which does not require any further local research approvals but does require the approval of the patient or parent.^52^ This web-based register is currently helping the EuroDSD research programme and has the potential to address many unanswered questions about long-term outcome in these rare conditions. Such registers of patients can also facilitate the development of local circles of patients and parents with similar conditions who can support each other.

## Conclusion

The rationale for investigating a newborn or an adolescent with a suspected disorder of sex development may include the need to determine the sex of rearing, anticipate early medical problems, explain the aetiology to the young person and the parents of an affected newborn and, finally, to develop a management plan that leads to optimal long-term outcome. A rational and empathic approach that relies on the skills and knowledge of the experts within the multidisciplinary team is essential for achieving these goals. An unequivocal diagnosis confirmed by biochemical and genetic means remains elusive in many cases of disorders of sex development, particularly XY disorders of sex development. The stepwise approach to reaching the final diagnosis needs to be explained to parents and the most important goals of the initial period of assessment should be to support the affected person and the parents, assign a sex of rearing and exclude the possibility of any early medical problems.

## Authors & Members of the Working Group

S. Faisal Ahmed: British Society of Paediatric Endocrinology & Diabetes, Society for Endocrinology, Chair & Corresponding Author

John C. Achermann: British Society of Paediatric Endocrinology & Diabetes, Society for Endocrinology

Wiebke Arlt: Society for Endocrinology

Adam Balen: British Society of Paediatric & Adolescent Gynaecology

Gerry Conway: Society for Endocrinology

Zoe Edwards: Chartered Member of the British Psychological Society

Sue Elford: CLIMB CAH Support Group

Ieuan A. Hughes: British Society of Paediatric Endocrinology & Diabetes, Society for Endocrinology

Louise Izatt: British Society of Human Genetics, Clinical Genetics Society

Nils Krone: British Society of Paediatric Endocrinology & Diabetes, Society for Endocrinology

Harriet Miles: British Society of Paediatric Endocrinology & Diabetes

Stuart O'Toole: Member of British Association of Paediatric Urologists

Les Perry: Association for Clinical Biochemistry, Society for Endocrinology

Caroline Sanders: Royal College of Nursing

Margaret Simmonds: AIS Support Group

A. Michael Wallace: Association for Clinical Biochemistry, British Society of Paediatric Endocrinology & Diabetes, Society for Endocrinology

Andrew Watt: British Society of Paediatric Radiology

Debbie Willis: Society for Endocrinology

## References

[1] Biason-Lauber A (2010). Control of sex development. Best Practice and Research. Clinical Endocrinology and Metabolism.

[2] Hughes IA, Houk C, Ahmed SF (2006). Consensus statement on management of intersex disorders. Archives of Disease in Childhood.

[3] Pasterski V, Prentice P, Hughes IA (2010). Consequences of the Chicago consensus on disorders of sex development (DSD): current practices in Europe. Archives of Disease in Childhood.

[4] Brain CE, Creighton SM, Mushtaq I (2010). Holistic management of DSD. Best Practice and Research. Clinical Endocrinology and Metabolism.

[5] Liao LM, Tacconelli E, Wood D (2010). Adolescent girls with disorders of sex development: a needs analysis of transitional care. Journal of Pediatric Urology.

[6] Karzakis K (2009). Fixing Sex: Intersex, Medical Authority, and Lived Experience.

[7] Karkazis K, Tamar-Mattis A, Kon AA (2010). Genital surgery for disorders of sex development: implementing a shared decision-making approach. Journal of Pediatric Endocrinology and Metabolism.

[8] Duguid A, Morrison S, Robertson A (2007). The psychological impact of genital anomalies on the parents of affected children. Acta Paediatrica.

[9] Cull ML, Simmonds M (2010). Importance of support groups for intersex (disorders of sex development) patients, families and the medical profession. Sex Development.

[10] Wiesemann C, Ude-Koeller S, Sinnecker GH (2010). Ethical principles and recommendations for the medical management of differences of sex development (DSD)/intersex in children and adolescents. European Journal of Pediatrics.

[11] Liao LM (2003). Learning to assist women born with atypical genitalia: journey through ignorance, taboo and dilemma. Journal of Reproductive and Infant Psychology.

[12] Bartlett YK, Coulson NS (2011). An investigation into the empowerment effects of using online support groups and how this affects health professional/patient communication. Patient Education and Counseling.

[13] Creighton SM, Minto CL, Liao LM (2004). Meeting between experts: evaluation of the first UK forum for lay and professional experts in intersex. Patient Education and Counseling.

[14] Ahmed SF, Dobbie R, Finlayson AR (2004). Regional & temporal variation in the occurrence of genital anomalies amongst singleton births, 1988–1997, Scotland. Archives of Disease in Childhood.

[15] Thyen U, Lanz K, Holterhus PM (2006). Epidemiology and initial management of ambiguous genitalia at birth in Germany. Hormone Research.

[16] Vidal I, Gorduza DB, Haraux E (2010). Surgical options in disorders of sex development with ambiguous genitalia. Best Practice and Research. Clinical Endocrinology and Metabolism.

[17] Ahmed SF, Khwaja O, Hughes IA (2000). Clinical features and gender assignment in cases of male undermasculinisation: the role for a masculinisation score. British Journal of Urology International.

[18] Moreno-Garcia M, Miranda EB (2002). Chromosomal anomalies in cryptorchidism and hypospadias. Journal of Urology.

[19] Boehmer ALM, Nijman RJM, Lammers BAS (2001). Etiological studies of severe or familial hypospadias. Journal of Urology.

[20] Creighton S, Alderson J, Brown S (2002). Medical photography: ethics, consent and the intersex patient. British Journal of Urology International.

[21] Shendure J, Ji H (2008). Next-generation DNA sequencing. Nature Biotechnology.

[22] Bashamboo A, Ledig S, Wieacker P (2010). New technologies for the identification of novel genetic markers of disorders of sex development (DSD). Sex Development.

[23] Chertin B, Koulikov D, Alberton J (2006). The use of laparoscopy in intersex patients. Pediatric Surgery International.

[24] Denes FT, Cocuzza MA, Schneider-Monteiro ED (2005). The laparoscopic management of intersex patients: the preferred approach. British Journal of Urology International.

[25] Chavhan GB, Parra DA, Oudjhane K (2008). Imaging of ambiguous genitalia: classification and diagnostic approach. Radiographics.

[26] Wright NB, Smith C, Rickwood AM (1995). Imaging children with ambiguous genitalia and intersex states. Clinical Radiology.

[27] Tomlinson C, Wallace AM, Ahmed SF (2004). Erroneous testosterone assay causing diagnostic confusion in a newborn infant with intersex anomalies. Acta Paediatrica.

[28] Tomlinson C, Macintyre H, Dorrian CA (2004). Testosterone measurements in early infancy. Archives of Disease in Childhood. Fetal and Neonatal Edition.

[29] Albrecht L, Styne D (2007). Laboratory testing of gonadal steroids in children. Pediatric Endocrinology Reviews.

[30] Nakamoto J, Fuqua JS (2007). Laboratory assays in pediatric endocrinology: common aspects. Pediatric Endocrinology Reviews.

[31] Krone N, Hughes BA, Lavery GG (2010). Gas chromatography/mass spectrometry (GC/MS) remains a pre-eminent discovery tool in clinical steroid investigations even in the era of fast liquid chromatography tandem mass spectrometry (LC/MS/MS). Journal of Steroid Biochemistry and Molecular Biology.

[32] Shackleton C, Marcos J, Malunowicz EM (2004). Biochemical diagnosis of Antley-Bixler syndrome by steroid analysis. American Journal of Medical Genetics.

[33] Kulle AE, Riepe FG, Melchior D (2010). A novel ultrapressure liquid chromatography tandem mass spectrometry method for the simultaneous determination of androstenedione, testosterone, and dihydrotestosterone in pediatric blood samples: age- and sex-specific reference data. Journal of Clinical Endocrinology and Metabolism.

[34] Teixeira J, Maheswaran S, Donahoe PK (2001). Müllerian inhibiting substance: an instructive developmental hormone with diagnostic and possible therapeutic applications. Endocrine Reviews.

[35] Ahmed SF, Keir L, McNeilly J (2010). The concordance between serum anti-mullerian hormone and testosterone concentrations depends on duration pf hCG stimulation in boys with disorders of sex development. Clinical Endocrinology.

[36] Grinspon RP, Rey RA (2010). Anti-müllerian hormone and sertoli cell function in paediatric male hypogonadism. Hormone Research in Paediatrics.

[37] Ahmed SF, Cheng A, Hughes IA (1999). Biochemical evaluation of the gonadotrophin-gonadal axis in androgen insensitivity syndrome. Archives of Disease in Childhood.

[38] Dixon JR, Wallace AM, O'Toole S (2007). Prolonged human chorionic gonadotrophin (hCG) stimulation as a tool for investigating and managing undescended testes. Clinical Endocrinology.

[39] Steinmetz L, Rocha MN, Longui CA (2009). Inhibin A production after gonadotropin stimulus: a new method to detect ovarian tissue in ovotesticular disorder of sex development. Hormone Research.

[40] Rosencrantz MA, Wachs DS, Coffler MS (2010). Comparison of inhibin B and estradiol responses to intravenous FSH in women with polycystic ovary syndrome and normal women. Human Reproduction.

[41] Krone N, Dhir V, Ivison HE (2007). Congenital adrenal hyperplasia and P450 oxidoreductase deficiency. Clinical Endocrinology.

[42] Shackleton CH, Blau N, Duren M, Gibson KM (2008). Genetic disorders of steroid metabolism diagnosed my mass spectrometry. Laboratory Guide to the Methods in Biochemical Genetics.

[43] Cameron FJ, Hageman RM, Cooke-Yarborough C (1996). A novel germ line mutation in SOX9 causes familial campomelic dysplasia and sex reversal. Human Molecular Genetics.

[44] Harley VR, Clarkson MJ, Argentaro A (2003). The molecular action and regulation of the testis-determining factors, SRY (sex-determining region on the Y chromosome) and SOX9 [SRY-related high-mobility group (HMG) box 9]. Endocrine Reviews.

[45] Biason-Lauber A, Konrad D, Meyer M (2009). Ovaries and female phenotype in a girl with 46,XY karyotype and mutations in the CBX2 gene. American Journal of Human Genetics.

[46] Lin L, Achermann JC (2008). Steroidogenic factor-1 (SF-1, Ad4BP, NR5A1) and disorders of testis development. Sex Development.

[47] Zhu YS, Imperato-McGinley JL (2009). 5alpha-reductase isozymes and androgen actions in the prostate. Annals of the New York Academy of Sciences.

[48] Lubahn DB, Brown TR, Simental JA (1989). Sequence of the intron/exon junctions of the coding region of the human androgen receptor gene and identification of a point mutation in a family with complete androgen insensitivity. Proceedings of the National Academy of Sciences of the United States of America.

[49] Bouvattier C, Carel JC, Lecointre C (2002). Postnatal changes of T, LH, and FSH in 46,XY infants with mutations in the AR gene. Journal of Clinical Endocrinology and Metabolism.

[50] Belgorosky A, Rivarola MA (1985). Sex hormone binding globulin response to testosterone. An androgen sensitivity test. Acta Endocrinologica (Copenhagen).

[51] Ahmed SF, Cheng A, Dovey LA (2000). Clinical features, androgen receptor binding and mutational analysis in 278 reported cases of the androgen insensitivity syndrome. Journal of Clinical Endocrinology and Metabolism.

[52] Ahmed SF, Rodie M, Jiang J (2010). The European DSD Registry – a virtual research environment. Sexual development. Sex Development.

